# Space Use and Movement Patterns in a Semi-Free-Ranging Herd of European Bison (*Bison bonasus*)

**DOI:** 10.1371/journal.pone.0147404

**Published:** 2016-02-03

**Authors:** Amandine Ramos, Odile Petit, Patrice Longour, Cristian Pasquaretta, Cédric Sueur

**Affiliations:** 1 Centre National de la Recherche Scientifique, Département Ecologie, Physiologie et Ethologie, Strasbourg, France; 2 Université de Strasbourg, Institut Pluridisciplinaire Hubert Curien, Strasbourg, France; 3 Réserve Biologique des Monts-d’Azur, Domaine du Haut-Thorenc, Thorenc, France; NYIT College of Osteopathic Medicine, UNITED STATES

## Abstract

The successful reintroduction and restocking of the European Bison demands a reliable knowledge of the biology of this species. Yet little is known to date about the European bison, and empirical data remains insufficient to set up a reliable plan ensuring the reintroduction, maintenance and survival of populations in habitats that have been largely modified by human activity. Studies of the ecology, social behaviour and management of bison are therefore crucial to the conservation of this species and its cohabitation with humans. To meet these challenges, we focused on movement patterns and space use in a semi-free-ranging herd of European bison living in the *Réserve Biologique des Monts-d’Azur* (France). Bison spend over 80% of their time foraging and resting; foraging mainly occurs around the artificial feeding sites (i.e., hay racks) or in meadows. The time of day and the presence of snow have no influence on the time budget allocated to each activity. Animals, however, spend more time at the food racks in winter. Bison also spend most of their time in small groups of individuals, confirming the occurrence of both fission-fusion dynamics and sexual segregation in this species. Bison seem to follow a Lévy walk pattern of movement, which is probably related to the geographical distribution and size of food patches in the reserve. The conclusions of this study provide a better understanding of the sociality, life habits and habitat use of bison, and also describe how the provision of hay affects all these behaviours. These results could be useful in the development of tools to select the most suitable habitats for the reintroduction, management and conservation of bison populations.

## Introduction

The disciplines of animal behaviour and behavioural ecology are essential tools for the implementation of reliable conservation measures [1, 2]. Currently, many species are put at risk by many threats that are often linked to human activity and the lack of knowledge concerning their biology makes it difficult to protect them [3]. The development of road and rail networks has increasingly fragmented and degraded natural habitats [4]. Populations are thus isolated from each other, impairing the genetic variability and as a consequence their survival [1]. We therefore need to understand the different social behaviours, movement patterns and habitat use of species if we wish to effectively predict the impact of environmental changes on populations and limit their effects on them [1].

Although large herbivores can have negative effects on the richness and availability of plant species, especially when they overcrowd their environment [5, 6, 7], they are also known to have a positive influence at both local and regional scales via local disturbances, selective grazing or even effective seed dispersion [8]. Despite this major role in the ecosystem, several large herbivores are endangered and registered on the IUCN Red List: in 2002, 84 of an estimated total of 175 ungulate species were critically endangered, endangered or vulnerable [8]. The European bison (*Bison bonasus*), the largest herbivorous mammal on the European continent [9], is one such species. Historically, it was distributed throughout western, central and south-eastern Europe, as well as the Caucasus [10]. Just two wild populations remained at the end of the 19^th^ Century: one in the Bialowieza forest (located on the border between Poland and Belarus) and the other in the western Caucasus Mountains [11]. Shortly after World War I, the species was considered extinct in the wild and the captive population consisted of just 54 specimens (29 males and 25 females). Thirteen of these surviving individuals were used for breeding purposes, hence creating a new basic genetic reservoir from which all the current populations of European bison were reconstituted [11, 12]. Despite increasingly numerous conservation efforts, European bison populations remain vulnerable, and are still listed as a protected species in the Berne Convention and the Habitat Directive [11].

As underlined by the European Bison Conservation Center, it is essential to gain a better understanding of the ecology of European bison through the study of their daily and seasonal activity, their movement patterns and their preferred habitats [11]. Although great progress has been made in this domain, the majority of studies carried out to date concerns bison populations in Białowieża Forest [13, 14], in regions of Eastern Europe [15, 16, 17, 18] or in the Carpathian Mountains [19, 20, 21]. It therefore appears essential to conduct systematic studies on populations located in areas further south in order to collect as much information as possible about bison ecology and life habits, in different habitats with different environmental conditions. This will not only improve the effective conservation and management of bison, but also their coexistence with humans, in particular by avoiding damage to agricultural and forestry plots after reintroduction through a better selection of the most suitable habitats for their biological needs [3, 22].

The impact animals can have on their environment is particularly dependent on their patterns of distribution and their abundance [23, 24, 25]. For example, the group size can influence their capacity to explore the environment and thus lead to group cohesion constraints [26]. Furthermore, differences between males and females often result in sexual segregation and fission-fusion dynamics in species showing high sexual dimorphism for nutrient requirements, activity budgets or exposure to predation [27, 28, 29, 30]. This fission-fusion dynamics, which can be also influenced by food availability, has been described in European bison [31], American bison (*Bison bison*) [25, 32] and African buffalo (*Syncerus cafer*) [33], but further studies are required to better understand its coevolution alongside environmental conditions (e.g. weather, habitat types, management choice, etc).

In this study, we focused on the daily activity of a semi-free-ranging herd of European bison in order to understand how its life habits and movement patterns change according to environmental and management conditions. European bison lives in herds composed of up to thirty individuals, and the average size and structure of these herds are environment dependent [11]. Groups can merge or split according to the season, the availability and distribution of resources or even the presence of predators [31, 34]. Mixed groups are composed of cows and calves with the temporary presence of adult males, while male groups contain about two adult males [31, 34]. However, more than half of the males are solitary and only join the main group during the rut period. Our main aim was to evaluate how the fission-fusion dynamics, habitat use and daily activity of our bison herd would be influenced by the provision of hay and by environmental conditions. When natural food resources become scarce during winter, the reserve places artificial feeding patches, dispersed across the territory. We would therefore expect bison to form separate groups of variable size around the artificial feeding sites during this season [11, 34, 35]. With the arrival of spring, the melting of snow gives better access to the grass and consequently increases the abundance of natural food resources. We would therefore expect to observe the bison regrouping in the meadow [34]. The animal movement patterns, namely Brownian or Lévy walks [36, 37, 38], would be expected to be mainly connected with feeding activity, playing an essential role in ensuring the optimum use of food resources. For many researchers, the Lévy walk is an optimal food search strategy used in heterogeneous environments with a low density of food patches, whereas the Brownian walk is mainly associated with the presence of abundant food resources [36, 38, 39, 40]. Our second objective was therefore to better understand the food search strategy of bison by studying their movement pattern. We hypothesize that bison use a Lévy walk movement given the relatively heterogeneous distribution of food patches, especially during the winter period.

## Materials and Methods

### Ethics Statement

The reserve has an approval to possess and breed European bison (certificate number: FR 00004165). This study was carried out by directly observing the animals, which were not subjected to any handling or invasive experiments. Our study was carried out in full accordance with the ethical guidelines of our institution (*Institut Pluridisciplinaire Hubert Curien*) and of the reserve, and complied with European animal welfare legislation. Every effort was made to ensure the welfare of the animals and minimize disturbance by the researchers.

### Study area

The study was carried out from 16 February to 26 April 2013 at the *Reserve Biologique* des *Monts-d’Azur*, located at the *Domaine du Haut-Thorenc* (43°48’20”N, 6°50’43”W), in the *Alpes-Maritime* region of France. This fenced reserve of about 700 hectares, located at an altitude of around 1200m, comprises seven different habitats: pine forest (51%, predominantly composed of Scots pine trees), meadow (33%, composed mainly of herbaceous vegetation, predominantly grasses), boxwood (12%), wet meadow (2%, grassed area flooded for part of the year), limestone grassland (2%, characterized by herbaceous perennial plants growing on a calcareous soil) and two water sites including an artificial lake. The *Reserve Biologique des Monts-d'Azur* is part of the original range of the European bison [11], and its climate and plant species make it suitable for the reintroduction of these animals.

### Study subjects

The semi-free-ranging herd of bison living on the reserve consists of 43 individuals: 3 adult males aged from 6 to 9 years, 13 sub-adult males aged from 1 to 3 years (one of which died in March 2013), 10 adult females aged from 7 to 13 years, and 8 sub-adult females aged from 1 to 3 years. The herd also includes 9 juveniles approximately one year old (3 males and 6 females). These bison arrived at the reserve in 2005 and 2006 following selection by the coordinator for the European Breeding Program for European Bison in order to ensure a natural sex ratio.

As the fenced area of the reserve is not large enough to provide sufficient resources during winter and given the possibility of deep snow covering the ground during this period, bison were supplemented with hay twice a week, from November 2012 to April 2013, to decrease their mortality rate and ensure their survival in the cold season. In particular, 10 racks were set up as sources of extra food (2 hay bales per rack). They were located along the edge of the pine forest from east to west.

### Data collection

We observed the bison herd for an average of 4 hours per day from 10 a.m. to 12 p.m. and from 4 p.m. to 6 p.m., for a total observation time of approximately 153h, with a comparable number of sessions in the morning (n = 42) and the afternoon (n = 41) for the entire study period. The observer (A.R.) was located approximately 20–25 m from the animals. This distance ensured that the animals, which were habituated to human presence, did not express stress behaviours [41]. Before each observation session, the outdoor temperature (°C) was evaluated using the thermometer of a DC360BL digital compass (Fotronic Corporation, Melrose, Massachusetts), and when snow was present, snow depth was measured in the meadow (average = 28.2±18.3, cm).

The daily activity of bison was recorded using the instantaneous sampling method [42] to collect the following parameters every 10 minutes: (1) the number of moving individuals, (2) the number of foraging individuals, (3) the number of standing individuals (with no specific activity), (4) the number of lying down individuals, (5) the number of isolated individuals (located more than 5 m from any congener), and (6) the number of individuals in social interaction. The social interactions that were taken into account included battling (usually males), suckling, social play and sexual behaviours. To better understand the social behaviour of bison, we also determined the dispersion state of the herd every 10 minutes. The bison were considered to be grouped when individuals were less than 5 m apart and dispersed when two thirds of the individuals were more than 5 m apart. When the minimum distance between individuals belonging to sub-groups was over 5 m (but individuals inside sub-groups were less than 5 m from each other) the state was considered as "sub-grouped." If the dispersion state did not correspond to one of the three states described above, the herd dispersion was considered as non-defined. This criterion of 5 m was estimated based on bison body length. A similar criterion was used for studies in other social ungulates species, especially cattle [43]. We noted the type of habitat (including hay rack sites) occupied by the majority of bison (i.e., the main group), and its GPS position (altitude, longitude and latitude). Finally, we used GPS points to study step length (distance over a period of 10 minutes, in km), the time between two successive movements (sec) and the angles between successive movements (degrees). Step lengths were calculated according to the interval between the coordinates of two consecutive points. Thus, the step length value was zero when there was no movement. The time between two movements (waiting time) was the time during which individuals had not moved (sum of same GPS points performed between two movements). The angle between two consecutive movements (turning angle) corresponded to the angle formed between the two vectors of successive movements, the latter being determined using three successive GPS points.

All the previous cited data were collected using Cyber Tracker 3.0 software (Cyber Tracker Conservation, Bellville, South Africa) in conjunction with a Personal Digital Assistant Trimble Juno^®^ 3B (Trimble Navigation Limited, Westminster, United-States).

### Statistical analysis

In order to meet our primary objective, i.e. to understand how additional feeding and environmental conditions influence the daily activity, habitat use and sociality of bison, we used Fisher’s exact tests to highlight any differences in the percentage of time spent in the different activities, the different frequented habitats, and the different states of dispersion of the herd during the morning and the afternoon and between weather conditions (i.e. the presence and absence of snow). For each type of activity, each type of frequented areas, and each state of dispersion, we ran pairwise comparisons between the two groups of conditions (i.e., morning vs. afternoon and “snow” vs. “no snow”) with Bonferroni corrections for multiple testing [44]. For each condition, Kruskal-Wallis tests were conducted to determine whether the time spent (% time budget) in each activity, habitat and state of dispersion was homogeneously distributed or not. If the test results were significant, Dunn multiple comparisons tests were run to determine which activities, habitats and states of dispersion differed significantly. These analyses were carried out on 993 scans.

To evaluate habitat use, all GPS points were used to estimate the distribution of the herd in the reserve throughout the study period using the Kernel Density Estimation method (R software, package adehabitatHR) [45].

We then analysed the sociodemographic factors of the herd. The Typical Group Size (TGS), which quantifies group size as experienced by the individual, was calculated as the sum of the squares of the number of individuals in each group, divided by the total number of animals sampled [46]. The TGS emphasises how the members of a population associate; this information is not revealed by the arithmetic mean of the size of the groups. The sex ratio of groups was calculated by dividing the number of males (adult and sub-adult) by the total number of adult and sub-adult individuals.

In order to assess how animals move in their territory and identify their foraging strategy, Kolmogorov-Smirnov testing was used to test the uniformity of angle distribution between two movements after correcting to allow for the use of this test on angles, i.e. absolute frequencies. A curve estimation test (linear, polynomial and exponential) was then performed to assess the movement distribution. We investigated results for functions that best explained the data distribution. Two hypotheses, the Lévy walk and the Brownian walk, were tested for step length distribution and time between two movements. The Lévy walk hypothesis is characterized by a power curve and distributions that show many short step lengths and some long step lengths; this pattern indicates some optimization of movements or food searching behaviour [36, 38]. The Brownian walk hypothesis is characterized by an exponential curve and by random and non-optimized movements. We tested for the predominance of either a Lévy walk (power distribution, equation 1: y = a.x^μ^) or a Brownian walk (exponential distribution, equation 2: y = a.exp^l.x^) in European bison. We checked the form of the distributions via the maximum likelihood method (MLE) [47, 48], which involves calculating the exponent of the distribution (i.e., power or exponential in the case of the current study) to calculate the log likelihood of the distribution. Log likelihoods for the exponential or power distribution can then be compared for different step lengths using the Akaike Information Criterion (AIC). One AIC value was calculated for each hypothesis (exponential or power), and we retained the hypothesis with the lowest AIC. A detailed method for calculating MLE and AIC are described in Sueur et al. [38]. AIC and the different estimates for parameters associated with power (exponent μ) and exponential (exponent *l*) were obtained using the fitdistr() and mle() functions respectively for the MASS and stats4 packages of the statistical software program R.

All statistical analyses were performed using R 3.0.1 software (R foundation for Statistical Computing, 2013, Vienna, Austria). For daily activity, habitat use and dispersion state, the histograms represent the mean ± standard error. The results of pairwise comparisons tests (between two conditions) are only shown in the figures when the Fisher’s exact tests are significant.

## Results

### Daily activity

**Fig 1:**
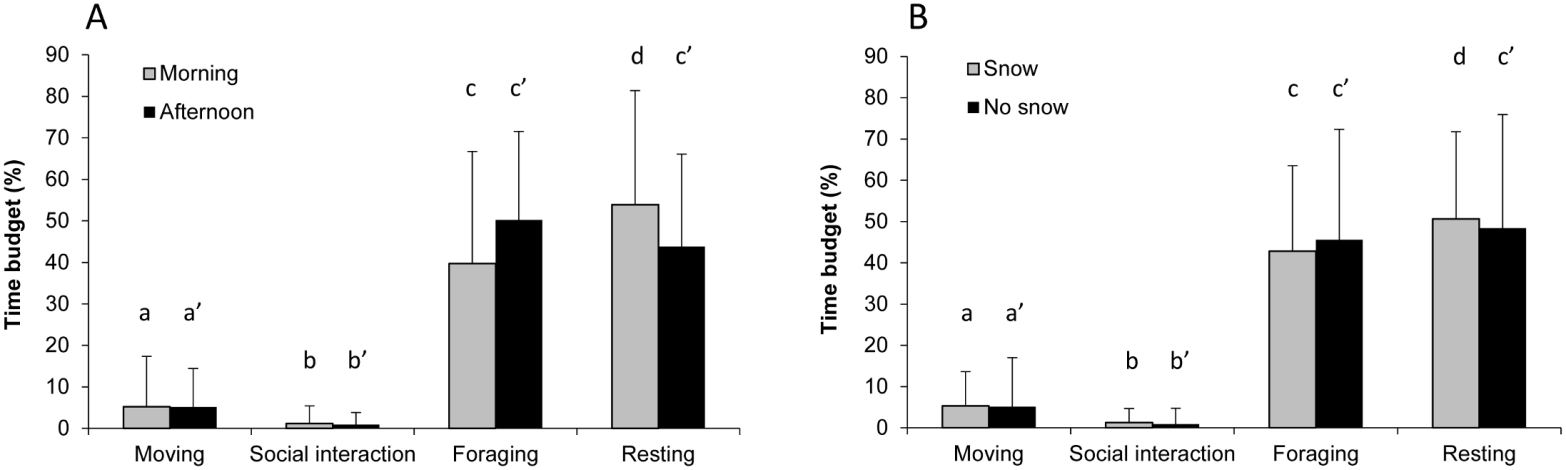
Group daily activity of bison (% time budget) according to the A) morning (grey bar) or afternoon (black bar) and B) presence (grey bar) or absence of snow (black bar). Different letters indicate significant intra-group differences between activities (*P* < 0.05) using Dunn's nonparametric multiple comparisons test.

No significant difference of bison activity was observed between the morning and the afternoon (Fisher’s exact test: *P* = 0.731). The time budget, however, is not homogeneously distributed among the different activity categories (Kruskal-Wallis tests: H_morning_ = 1326, df = 3, *P* < 0.001; H_afternoon_ = 1364, df = 3, *P* < 0.001, Fig 1A). In the morning, bison spent significantly more time in resting activity than foraging, moving and being involved in social interactions (Dunn's nonparametric multiple comparisons test, *P* < 0.05). We obtained the same tendency for the afternoon, but with equal amount of time spent foraging and resting.

Although the presence or absence of snow was not seen to make any significant difference to bison activities (Fisher’s exact test: *P* = 0.957), the time budget allocated to each activity is not uniformly distributed (Kruskal-Wallis tests: H_snow_ = 910, df = 3, *P* < 0.001; H_no snow_ = 1756, df = 3, *P* < 0.001, Fig 1B). Indeed, when there was snow, individuals spent more time resting than foraging, moving, and displaying social interactions (Dunn's nonparametric multiple comparisons test, *P* < 0.05). In the absence of snow, we observed that the time spent foraging was equivalent to the time spent resting.

### Habitat use

**Fig 2:**
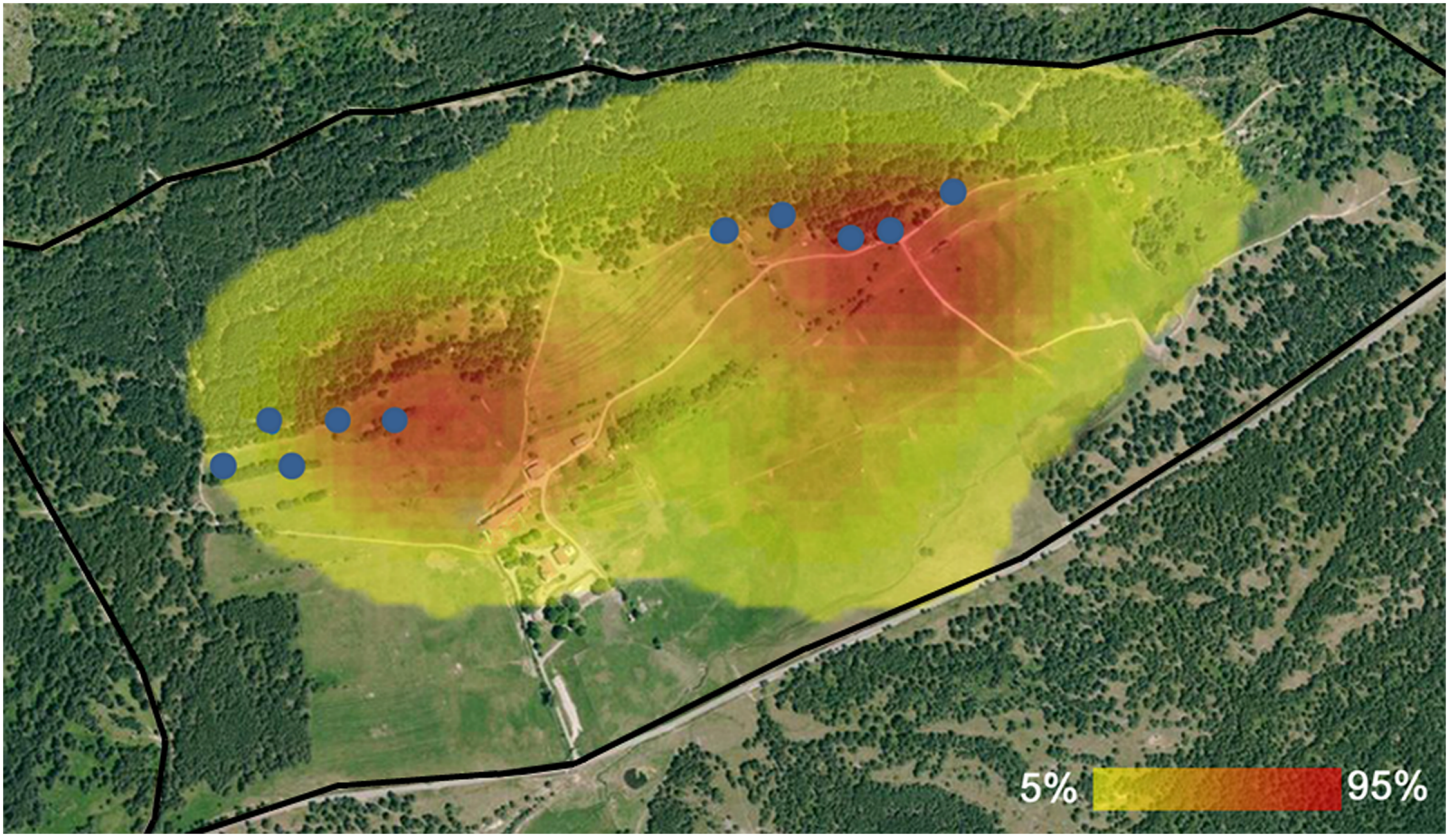
General occupation of bison in the *Monts-d’Azur* reserve (density by kernel density estimation) for the whole period of study. Areas of high density are red and those of low density are yellow. The blue dots represent the different racks and the black line corresponds to the fence of the reserve. Reprinted from BD ORTHO^®^ 50 cm under a CC BY license, with permission from © IGN, original copyright 2015.

The overall distribution of animals throughout the study period as determined by kernel density estimation shows that bison spent more than 50% of their time at the feeding racks (core area) and in the plots of meadow located nearby (Fig 2).

**Fig 3:**
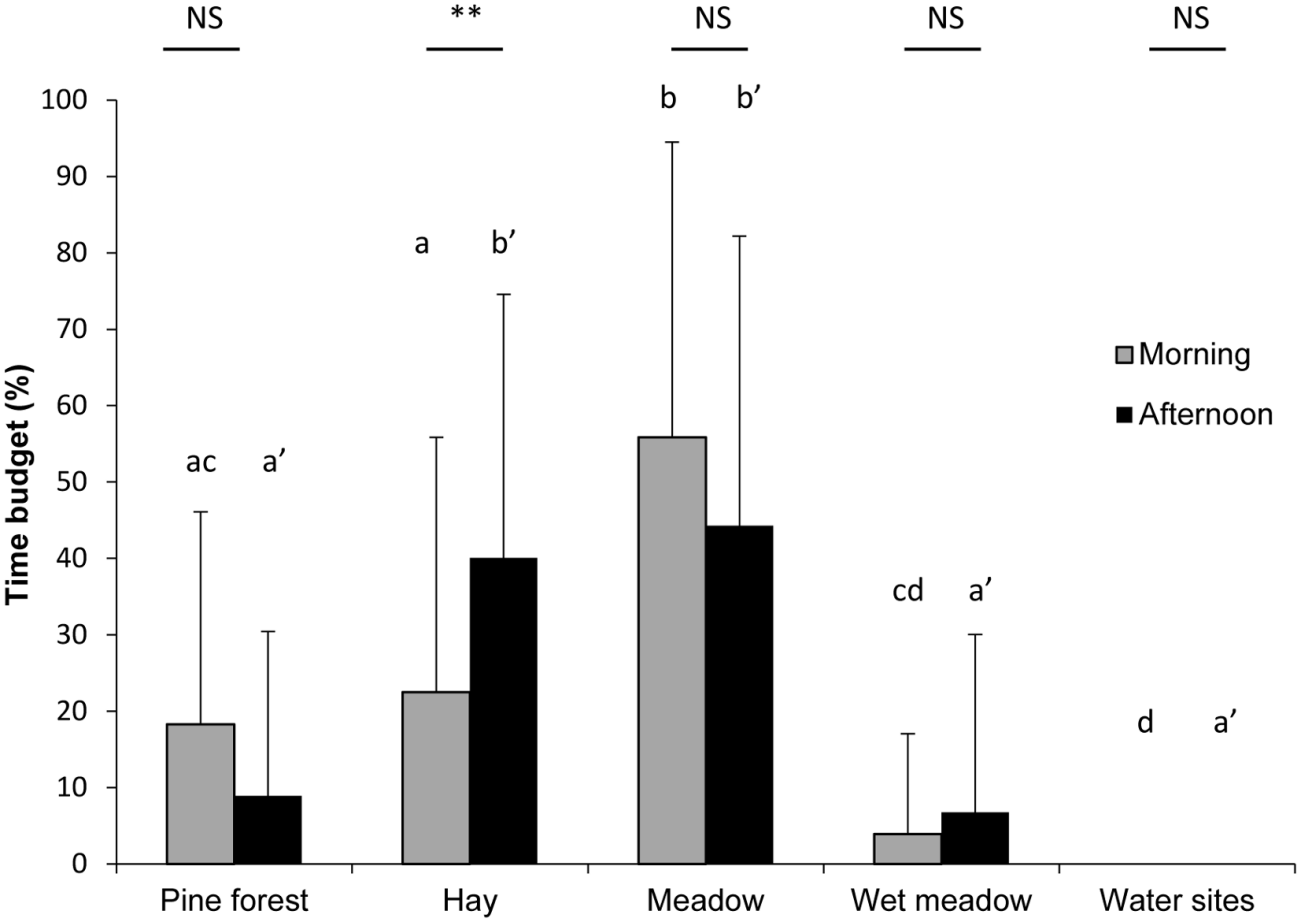
Habitats frequented by bison (% time budget) in the morning (grey bar) or the afternoon (black bar). NS: *P* > 0.05; **: *P* < 0.01. The presence of one same letter indicates that there is no significant intra-group difference between habitats (*P* > 0.05) using Dunn's nonparametric multiple comparisons test.

One significant difference in the habitats frequented by bison between the morning and the afternoon (Fisher’s exact test: *P* < 0.001, Fig 3) is that individuals visited the feeding racks significantly more often during the afternoon than in the morning (Mann-Whitney test: U_hay_ = 66.50, n_morning_ = 42, n_afternoon_ = 41, *P* ≤ 0.006). No significant difference in habitat use was observed for the other habitats (Mann-Whitney tests: U < 861, n_morning_ = 42, n_afternoon_ = 41, *P* > 0.083). In addition, the time spent in each habitat is not homogeneously distributed, either in the morning or the afternoon (Kruskal-Wallis tests: H_morning_ = 79, df = 4, *P* < 0.001; H_afternoon_ = 83, df = 4, *P* < 0.001). In the morning, the animals spent significantly more time in the meadow than in the pine forest, at the feeding racks, in the wet meadow or at the water sites (Dunn's nonparametric multiple comparisons test, *P* < 0.05). During the afternoon, individuals were mainly observed close to the racks and in the meadow rather than in the pine forest, the wet meadow, or at the water sites (Dunn's nonparametric multiple comparisons test, *P* < 0.05).

Bison used different habitats according to “snow” or “no snow” conditions (Fisher’s exact test: *P* < 0.001). They were more frequently observed near the feeding racks in the presence of snow (Mann-Whitney test: U_hay_ = 152, n_snow_ = 16, n_no snow_ = 32, *P* = 0.023). Concerning the other habitats, no significant difference was found between the “snow” and “no snow” conditions (Mann-Whitney Tests: U < 248 = 202, n_snow_ = 16, n_no snow_ = 32, *P* > 0).

### Dispersion state

**Fig 4:**
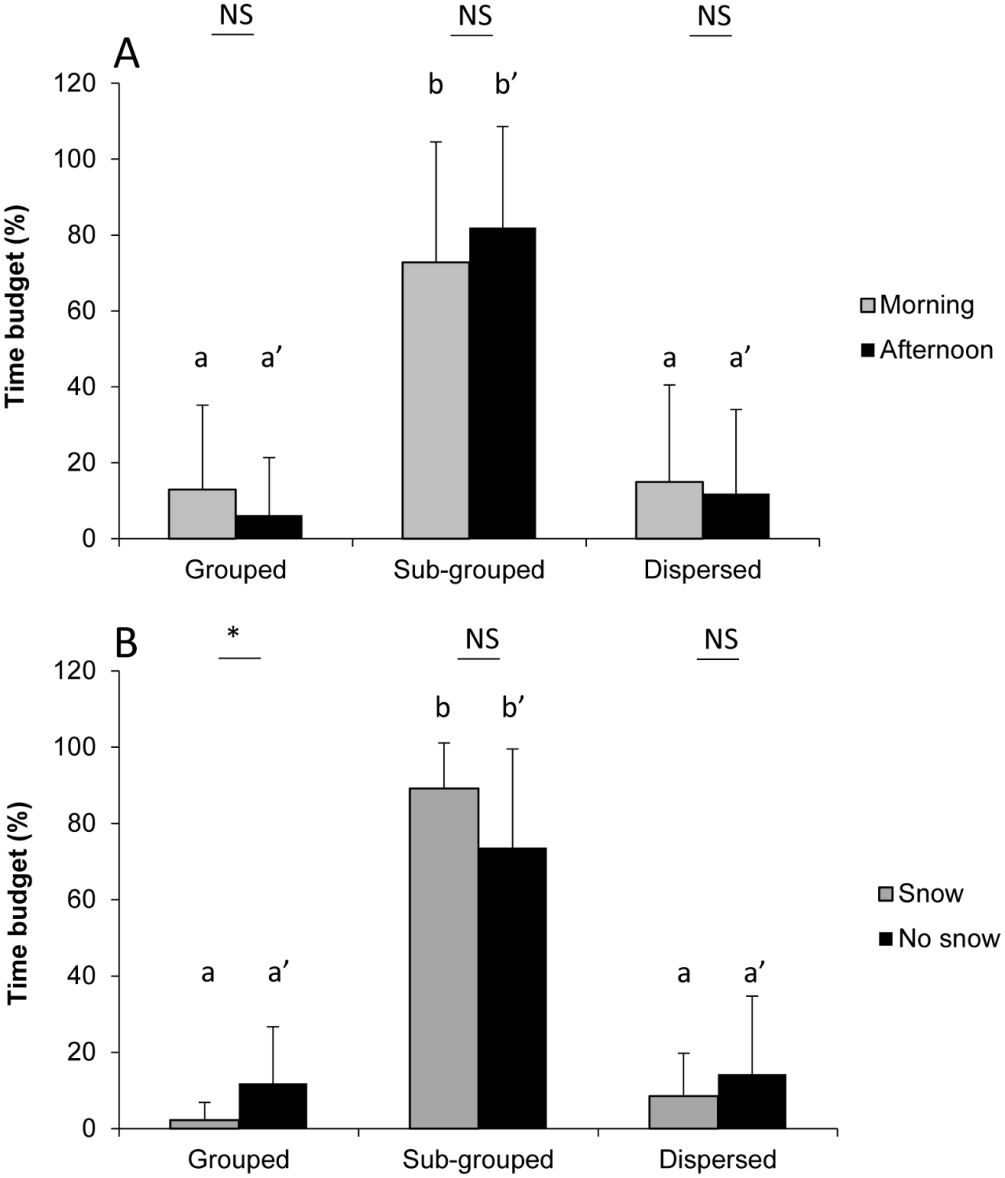
Dispersion states of bison (% time budget) according to A) the morning (grey bar) or the afternoon (black bar) and B) presence (grey bar) or absence of snow (black bar). NS: *P* > 0.05; *: *P* < 0.05. Different letters indicate significant intra-group differences between dispersion states (*P* < 0.05) using Dunn's nonparametric multiple comparisons test.

The time spent in each state is not distributed homogeneously during the day (Kruskal-Wallis tests: H_morning_ = 57, df = 2, *P* < 0.001; H_afternoon_ = 82, df = 2, *P* < 0.001): bison spent more time in sub-groups (70 to 80% of their time) than in “dispersed” and “grouped” states (Fig 4A). We found no difference between the morning and the afternoon (Fisher’s exact test: *P* ≤ 0.153). However, the states of dispersion differ between the “snow” and “no snow” conditions (Fisher’s exact test: *P* < 0.001, Fig 4B). The animals were more often grouped when there was no snow than in the presence of snow (Mann-Whitney test: U_grouped_ = 169, n_snow_ = 16; n_no snow_ = 32, *P* = 0.035) but no significant difference was observed between these two conditions for the “sub-grouped” and “dispersed” states (Mann-Whitney tests: U < 248, n_snow_ = 16; n_no snow_ = 32, *P* < 0.110).

### Typical group size and sex ratio

**Fig 5:**
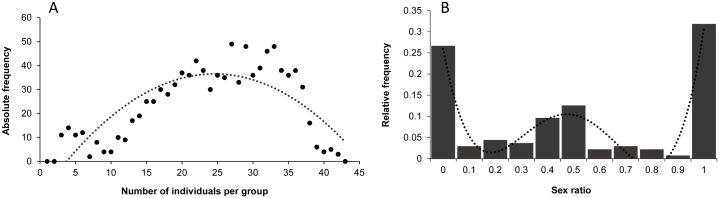
A) Number of individuals per group (absolute frequency) and B) sex ratio of groups (relative frequency). The dots in (A) correspond to the observed data and the dotted line in (B) corresponds to the polynomial curve that best fits the observed data.

Bison formed groups of highly variable sizes over time (Fig 5A) with an average of 24.9 ± 8.8 individuals. The distribution of group sizes follows a parabolic function (R² = 0.7, F_3,42_ = 81.8, *P* < 0.001, y = -0.084x^2^ + 4.119x - 14.214) and does not follow a normal distribution (t = 9.042, *P* < 0.001). Groups composed of low and high numbers of individuals were rarely observed compared to groups of intermediate size. The TGS was 28 individuals. High sexual segregation was revealed, since the distribution best follows a polynomial function of degree 4 (R² = 0.9, F_1,9_ = 139, *P* < 0.001, y = 0.0012x^4^–0.028x^3^ + 0.221x^2^–0.693x + 0.759) showing three peaks (Fig 5B). We observed higher frequencies for exclusively male (sex ratio = 1) and female (sex ratio = 0) groups. Mixed groups were also observed, but with intermediate frequencies (average relative frequency: 0.05 ± 0.04), indicating a fission-fusion phenomenon.

### Patterns of movements

**Fig 6:**
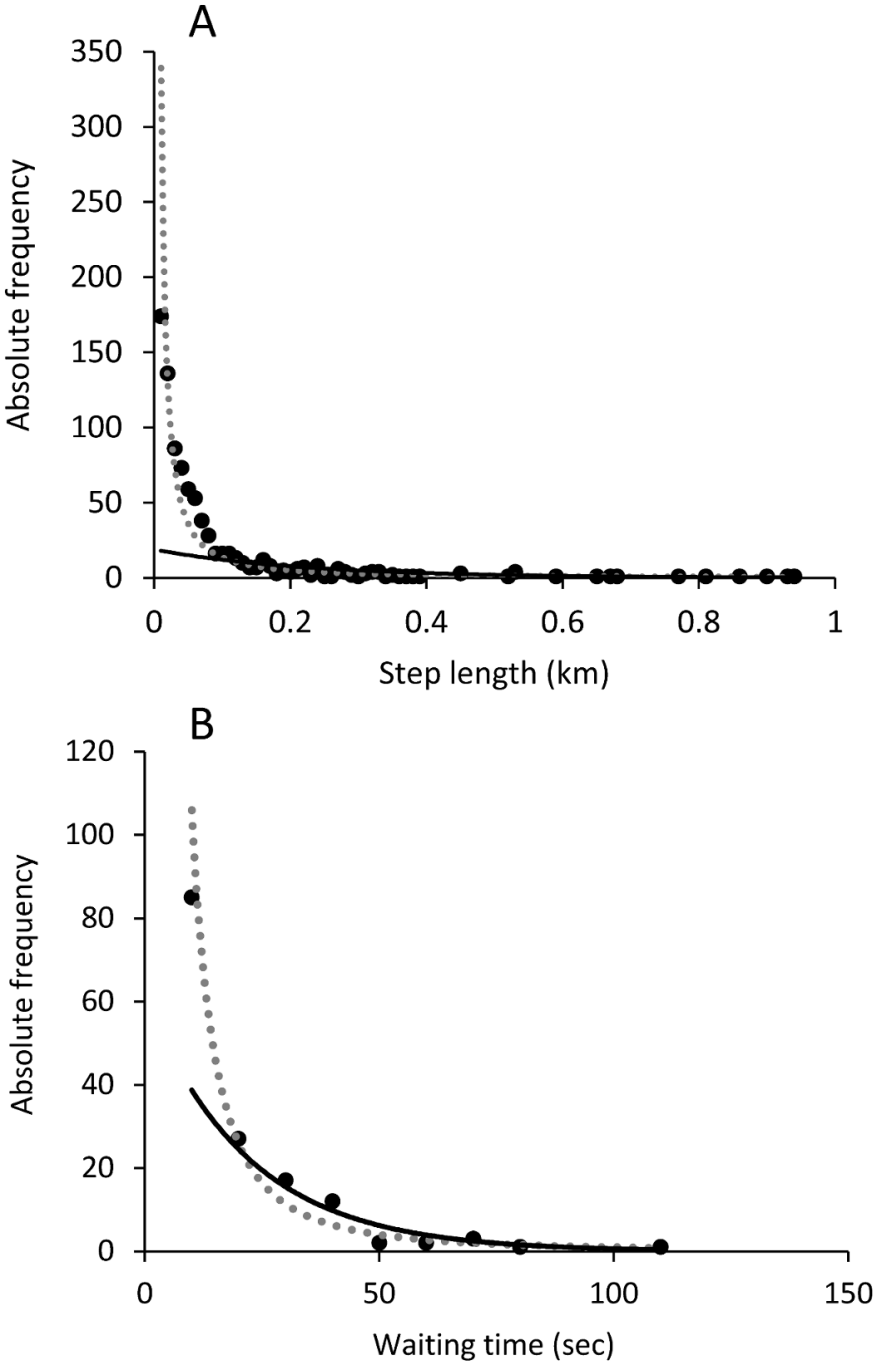
Distribution (absolute frequencies) of A) step lengths (km) and B) waiting time (sec). The dots correspond to the observed data, the continuous line to the exponential curve best fitting the data and the dotted line to the power curve best fitting the observed data. The model that best explains distributions of data for both (A) and (B) is the Lévy walk (power law).

The distribution of step lengths (absolute frequencies, km) follows a power function better than an exponential function (R²_power_ = 0.9, R²_exponential_ = 0.6, AIC_power_ = 5482.16 < AIC_exponential_ = 9208.33, y = 0.546x^-1.396^, y = 18.864e^-4.429x^, Fig 6A). Individuals seem to follow a Lévy walk movement rather than a Brownian walk movement type. Similarly, the distribution of stationary time between two movements (sec) follows a power function (R²_power_ = 0.9, R²_exponential_ = 0.8, AIC_power_ = 727.95 < AIC_exponential_ = 992.78, y = 11414x^-2.032^, y = 61.217e^-0.046x^, Fig 6B).

**Fig 7:**
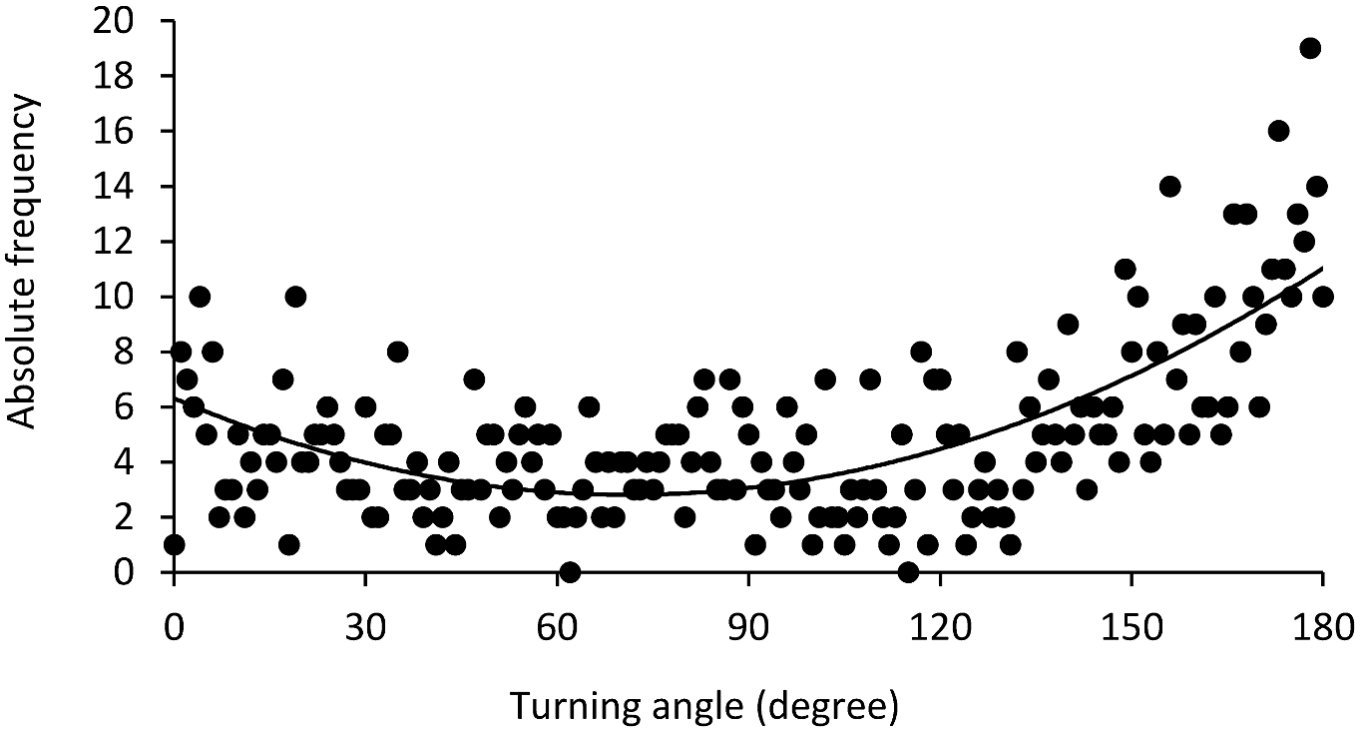
Distribution of angles (measured in degree) between two movements. The dots represent observed data and the line shows the best function explaining the distribution of degrees. Here, the function is parabolic, indicating that movements are goal directed.

The distribution of the angles between two movements (measured in degrees) is not uniform (Kolmogorov-Smirnov test: Z = 6.27, *P* < 0.001). Indeed, it follows a parabolic function (R² = 0.53, F_1.180_ = 65.21, *P* < 0.001, y = 0.0007x² - 0.098x + 6.310, Fig 7), with a higher frequency for angles close to 0° and 180°.

## Discussion

The main goal of this study was to acquire a better understanding of the movement patterns and space use of European bison according to the provision of extra food resources and environmental conditions. We therefore focused on the daily activity of individuals, their preferential use of habitat, the dispersion state of the herd and the pattern of their group movements. This data will provide a database that is essential to improving the reintroduction and the long-term management of European bison in Europe reserves.

We first studied the daily activity of bison by comparing the time budget for various activities between the morning and the afternoon and between "snow" and "no snow" conditions. We found that individuals spent most of their time resting and foraging, while they invested less time moving or interacting. These results are confirmed by literature showing that the daily activity of European bison is mainly characterized by the alternation of resting and foraging phases [35, 49]. Similar results have also been found for American bison where these two activities represented on average 87.9% of the time budget of a group [50]. In domestic cattle, domestic goat (*Capra aegagrus hircu*) and the chamois (*Rupicapra rupicapra*), foraging and resting phases represent 89%, 80% and 92% respectively of their daily time budget [51]. This pattern of activity seems to be observed in the majority of ruminants [35, 52] where the resting and foraging phases are interspersed by some movements allowing individuals to change sites to forage and rest or to avoid predators [46, 51].

The activity budget we observed was maintained both in the presence and the absence of snow cover, with a greater time spent resting than spent foraging in the presence of snow. Our results are somewhat different to findings by other studies [11]. Indeed, Caboń-Raczyńska et al. found that European bison allocated approximately 60% of their time budget foraging and 30% resting during winter grazing, while they observed the contrary during summer grazing [35, 49]. A similar seasonal influence on these two behaviours has also been described for American bison, for which the time spent foraging increased from summer to winter while resting time decreased [53]. These findings conflict with our results. This, and the lack of notable difference between the presence and the absence of snow in our study, could be explained by the observation period, limited to winter and early spring, and also by the food supplement that bison received during a large part of the study. Indeed, the supplementary fodder provision may affect some natural behaviours, especially those linked to foraging. This is confirmed by Rutley et al., who showed that the foraging and resting cycles of American bison were less distinct with food supplement [53]. Caboń-Raczyńska et al. have also shown that winter supplementary fodder provision caused an increase in resting time and a decrease in foraging [35, 49]. Finally, snow cover and food supplementation can also be responsible for the low mobility of bison during winter [34]. Indeed, our results are similar to those of Caboń-Raczyńska et al., for which bison spent on average 10% of their winter time roaming when their food was supplemented [35, 49].

Bison frequented different habitats in the morning and the afternoon. Individuals were observed more often at the hay racks in the afternoon than in the morning. It is interesting to note that the meadow was one of the busiest habitats, while individuals were rarely observed at water sites whatever the period of day. The general distribution map of animals shows that they predominantly attended the supplied hay sites and surrounding areas of meadow. Our results can mainly be explained by the provision of hay, which was more frequent in the afternoon than in the morning. The influence of food provision seems to be confirmed when the snow melts and the food supply decreases and stops; animals then spent significantly less time near the racks and were mainly observed in the meadow. This is unusual because the European bison is often described as a forest species showing a preference for deciduous or mixed forests [22, 54]. However, recent studies suggest that the European bison originally lived in relatively open areas and that its survival in our contemporary forests would therefore be an adaptation to environmental changes and human pressure [3, 55, 56]. This would define the European bison primarily as a grazer living in a suboptimal habitat [3, 56].

The majority of our data was collected over the winter season and revealed the use of snow by bison for their water needs, hence explaining the low attendance of water sites. This type of behaviour has already been observed in this species [11, 35] and in American bison [41]. Additionally, the fact that melting snow saturated the meadow with water during spring probably had a negative influence on the presence of bison at the lake.

By studying the state of dispersion of the herd, we showed that bison spent the majority of their time in sub-groups regardless of the conditions. The results could be explained by the distribution of the racks, which were distributed throughout the reserve and formed separate feeding sites. However, previous studies of the European bison led to similar results. Individuals have been found to form mixed groups of variable size (according to the period of year) and small peripheral groups of males [31, 34]. The presence of this organization in the American bison [41, 57] and in many species of cattle and deer [58] also supports our results. Furthermore, previous studies described how these groups frequently meet and split, with some individuals changing groups according to the season [58]. This fission-fusion dynamics is observed in many social species; when food resources are limited or unpredictable, groups often divide and decrease competition. The fusion or fission of groups can also be a response to predation pressure or individual nutrient requirements, which can lead to sexual segregation [59]. This phenomenon is particularly pronounced in some ungulate species, in which males are considerably larger than females and are therefore less vulnerable to predation and have higher nutritional needs [30]. Thus, males and females often move in separate groups, except during the breeding season, when the nutritional requirements does not differ so much between males and females due to the gestation and feeding of calves [30]. Sexual dimorphism is not the only hypothesis proposed in the literature to explain sexual segregation, which can be also explained by innate preference for same-sex peers [60] or sexual differences in activity budgets [30]. For some species, the grouping of congeners with similar needs remains a way to minimize the possible costs of synchrony [61].

We finally analysed group movement patterns to investigate whether bison optimize their use of food resources. The distribution of step lengths (km) and time between two movements followed a power function, meaning that bison use a Lévy walk movement pattern. It is characterized by a high number of small movements and few long movements. Indeed, bison made a lot of small movements around the principal food sites (racks) and made longer movements to move from one rack to another because racks were dispersed throughout the reserve. The distribution of data concerning the angles between two movements follows a parabolic curve (many values of 0° and 180°), indicating that bison perform linear movements with many u-turns. The linearity of movements is probably also related to the location of hay provision sites, with animals moving mainly from one rack to another and making very few small movements in the meadow or the forest.

Studying how bison use their habitat via the observation of herd distribution and the evaluation of impacts this species has on the environment could be key elements in the creation of measures for their protection and the improvement of the cohabitation with humans. Conflicts with human populations result in one of the greatest threats to the persistence and survival of many animal species in the wild [22, 62]. Human pressure on the natural environments pushes animals out of their territories, often resulting in damage to industrial and agricultural lands [22, 56]. The development of artificial food patches in natural habitats to keep animals away from private lands is a possible solution to reduce the conflicts between human activities and wildlife [22]. This method has been tested in South Africa to keep chacma baboons (*Papio ursinus*) away from urban areas [63]. Artificial food patches influence the ranging behaviour of species [64, 65] and are therefore attractive management tools to help prevent animal populations from leaving their natural range and dispersing [22]. Our study clearly shows that bison remain close to the racks when supplied with hay. However, supplementary feeding alone cannot represent a long-term solution, because it causes bison aggregation and leads to higher parasitic transmission, negatively affecting body condition [66, 67]. This study also reveals new elements that contribute to our understanding of space use and movement patterns in bison. Kernel estimation allows to indicate the surface needed by the population when food supply is present. Combined with other studies, these results can help to evaluate the surface necessary for a herd to live and survive without human intervention. However, as the majority of European reserves are totally fenced in, alternative solutions to decrease population size increase should be found. Our ultimate goal would be to predict all these elements to efficiently reintroduce this emblematic species in the most favourable habitats, which are now mostly anthropogenic.
